# Substance use and associated factors among Debre Berhan University students, Central Ethiopia

**DOI:** 10.1186/s13011-018-0150-9

**Published:** 2018-04-05

**Authors:** Tesfay Birhane Gebremariam, Kalayu Birhane Mruts, Tedla Kutaye Neway

**Affiliations:** 1 Department of Public Health, College of Health Sciences, Debre Berhan University, PO.Box. 445, Debre Berhan, Ethiopia; 2 Department of Psychology, College of Humanities and Social Sciences, Debre Berhan University, Debre Berhan, Ethiopia

**Keywords:** Substance use, Illicit drugs, University students, Debre Berhan

## Abstract

**Background:**

Being a global burden of youths, substances use is unhealthy behavior that exposes youths to health and social problems. Knowledge of the prevalence and predictors of substance use behavior among university students is important for designing periodic and locally appropriate interventions. This study is conducted to assess the prevalence and predicators of substances among Debre Berhan University students.

**Methods:**

Cross-sectional quantitative study was employed in May 2016. Stratified two-stage sampling technique was applied to choose 695 students. Substance use behaviors were assessed using tools derived from World Health Organization Model Students’ Substance Use Core Questionnaire.

**Result:**

The lifetime utilization of alcohol, khat and cigarette among students was found to be 36.3%, 10.9% and 7.4% respectively. The lifetime utilization of shisha and cannabis was 4.2% and 4.5% respectively. About 17%, 5.7%, and 3.1% of students are currently using alcohol, Khat and Cigarette respectively. Using multivariate binary logistic regression, being male, feeding out of the university café, being from private preparatory school, having higher monthly income, having substance user families and friends were found to be variables significantly associated with students’ substance use behaviors.

**Conclusions:**

The current prevalence of substances use among Debre Berhan University students is low comparing to other Ethiopian and African universities. Youth are starting substance use at lower grades especially at preparatory schools. Substance use behaviors are affected by complex factors at individual, family, school, social, and environmental factors. Therefore, strategies to alleviate youth substance use problems should focus on changing individual perception, knowledge, and intention towards substances. There is a need for further research with more powerful sample size and weighted estimates using complex analysis. Reasons for lower prevalence of substance use from other Ethiopian universities shall be further explored using qualitative study.

## Background

Drug use, drug use disorders and related health conditions are major public health concerns. According to WHO’s estimates for 2015, psychoactive drug use is responsible for more than 450,000 deaths per year. The drug-attributable disease burden accounts for about 1.5% of the global burden of disease, and injecting drug use accounts for an estimated 30% of new HIV infections outside sub-Saharan Africa and contributes significantly to the epidemics of hepatitis B and hepatitis C in all region [1]. The global burden of disease attributable to alcohol and illicit drug use is significant, accounting 5.4% of the total burden of disease in 2009. Another 3.7% of the population global burden of disease is attributable to tobacco use [2]. It is estimated that 9% of the global population aged 12 or older are classified with dependence on psychoactive substances such as alcohol [3].

Alcohol, khat, cigarette, hashish, and other illicit drugs like cannabis and cocaine are among the most used substances globally. In 2010, 38.3% of the global population consumed alcohol. In 2012, about 3.3 million deaths or 5.9% of all global deaths were attributable to alcohol consumption, 139 million DALYs (disability-adjusted life years) or 5.1% of the global burden of disease and injury were attributable to alcohol consumption [4]. In 2013, 21% of adults globally were current smokers – 950 million men and 177 million women. Tobacco kills up to half of its users accounting more than 7 million people each year. More than 6 million of those deaths are the result of direct tobacco use while around 890,000 are the result of non-smokers being exposed to second-hand smoke. Nearly 80% of the world’s more than 1 billion smokers live in low- and middle-income countries [5].

The use of alcohol, khat and tobacco among adolescents can be harmful, leading to increased health problems, decreased academic performance, reduced productivity, hopelessness and increased risky sexual behaviors [6, 7]. Many young people are also suffering from lack of self- esteem and future hope, victims of different forms of violence and abuse, or obliged to live with harmful habits like smoking, drug abuse and alcoholism [8]. Despite their large proportion and huge economic potential, adolescents and youths are vulnerable to variety of psychological, physical, social and sexual risky behaviors. Consumption of licit and illicit substance and its multidimensional consequence is one of the current challenges of young population group. Currently, significant numbers of youths are located in higher institutions where they are exposed to different new risky behaviors. Beside the individual biological and psychological vulnerability, peer pressure, social, environmental, academic pressures and being free of their parental influences may aggravate university students’ engagement to risk behaviors like substance use.

Researches indicated that the use of substances among young population is high. Substance use survey in Sudan reported an overall prevalence of substance use as 31%. The study reported current prevalence of tobacco, cannabis, cocaine, and heroin use at 13.7%, 4.9%, 0.7%, and 0.5% respectively. Being a male student was the principal predictor for substance use [9]. Lifetime prevalence rate of any substance use in Kenya was reported as 69.8% for alcohol, 51.9% for cigarette, 42.8% for cannabis, and 2% for cocaine). Males having statistically significantly higher rates than females [10].

The 2016 Ethiopian Demographic and Health Survey showed that 35% of women and about half of men (46%) reported drinking alcohol at some point in their lives. It also reported that 12% of women and 27% of men report having ever chewed chat. About 4.2% of men aged 15–59 years and 0.6% of women aged 15–49 years are cigarette smokers. Smoking in younger men population (age 20–24 years) is as low as 2.6% [11]. The 2015 Ethiopian national STEPS survey on risk factors for non-communicable disease reported that 4.2% of the survey participants were current (30 days) smokers. Only 3.1% young people aged 15–29 are current cigarette smokers. The survey revealed that, over all, mean age of smoking started among smokers was 21 years [12]. With regard to alcohol consumption, nearly 41% had consumed alcohol during the past 30 days prior to the survey. About 36.6% of young people aged 15–29 years are current alcohol users. The proportion of men who consumed alcohol (46.6%) was higher than that of women (33.5%). The survey also reported that 16% of respondents were current khat chewers and 7% of current khat chewers drank alcohol while using khat [12].

Of the young segment of the Ethiopian population, college and university students are at risk of such problems such as alcohol, khat and tobacco abuse. The lifetime prevalence of alcohol drinking, khat chewing, and cigarette smoking among Axum university students were 34.5%, 28.7% and 9.5% respectively. Similarly, the current prevalence of khat chewing, alcohol drinking and cigarette smoking were 27.9%, 32.8% and 9.3% respectively. The commonest reasons for khat, alcohol and cigarette using were to keep alert while reading, for relaxation and to relief stress respectively. Having peer friends who chew khat and family members who use alcohol were strongly associated with substance use [13]. A study among Hawassa university students reported lifetime and last 30 days substance use prevalence of 53.6% and 35.5% respectively. The rate of poly drug use (two or more PASs) was 18.3% [14]. Another study in the same university reported current (last 30 days) prevalence of khat chewing at 11.1% [15]. The study also showed living off-campus in rented houses and having friends who chew khat were found to significantly increase the odds of current regular khat use.

A study among Bahirdar University students reported twelve month and one month khat use of 12.7% and 7.7% respectively. Male students were more likely to chew khat in the current academic year as compared to female students. Students who live in off campus housing, have close friends who are khat chewers and family history of khat chewing were more likely to chew khat than their counterparts [16]. Other similar studies conducted in different Ethiopian Universities also reported students’ alcohol consumption rate ranging from 17% to 36%, Khat chewing from 7% to 33%, and smoking from 9% to 21% [17–20].

In general, the above studies indicated that substance use behavior and its risk factors varies across different universities. Production and customization of substances, especially Khat, by the nearby community may also greatly affect students’ utilization of substances. Majority of researches among the Ethiopian universities were done on alcohol, khat and cigarette leaving the illicit drugs like cannabis and cocaine. Therefore, having the above reports of substance use in different senior Ethiopian universities, it is vital to assess the prevalence and predictors of licit and illicit drug use among the newly established universities like Debre Berhan University for which this study is designed.

## Methods

A cross sectional quantitative study was conducted in June 2016. The study was conducted in Debre Berhan University (DBU) found 130 Km North of Addis Ababa. Being one of the newly established (second generation) universities, DBU has 10 colleges with 40 departments. There were a total of 11, 864 (4, 339 female & 7525 male) regular students enrolled in 2015/16 academic year and were considered as study population. Sample size was calculated using Epi-info version 3.5 with 29% prevalence of substance use [13], 95% confidence interval, 5% marginal error, design effect of 2 and 10% of non-response rate. Then the final sample size became 695.

Two stage stratified sampling technique was used to choose these students across different departments. First, students were stratified to their specific colleges to which the total sample size was distributed proportionally. Based on the sample size in each college, one or more departments were selected from each college using lottery method. The total sample size in the college was distributed to the selected department/s and allocated to each year of study. Finally, questionnaire was distributed to students in the class room starting on the right hand front corner of the class and orderly jumping one chair (Systematic Sampling Technique) until the required sample size for the section is achieved.

### Data collection tools and measurements

Substance use was assessed using items derived from the World health Organization (WHO) Model Students’ Substance Use Core Questionnaire [21]. This core questionnaire contains sections on socio demographic, smoking, alcohol and other drugs uses. We assess only substance that are commonly known in Ethiopia (alcohol, khat, tobacco, cannabis, cocaine and heroin). Other substances included in the WHO core module like tranquillizers, sedatives and hypnotics, amphetamines or other stimulants, opium, opiate drugs, volatile inhalant and injected drugs were not included in the survey. As per the WHO guide on substance abuse epidemiology, the nonexistent drug “Relevin” was included as a “validity check” to ensure that students did not over estimate their drug use [21]. Substance utilization behavior was asked both for life time and current (last 30 days).

In Ethiopia, there are many religious and other social festivities that were celebrated using local alcohols. Most people, including those who didn’t like to drink or are not regular drinkers, consume a small amount of alcohol during this religious and social events. For example during religious events, they test the local alcohol just to be blessed (locally called ‘Tsebel mekmes’) from the religious event even if they are not alcohol consumers. Therefore, in order to get best estimate of regular alcohol drinkers, consumption was asked for regular use by asking the question “have you consumed alcohol in the last 30 days except tasting for religious and social ceremonies?” Additional questions on perceived effects of substances, time to start substance use, reasons for substance use, and variables of substance use were also included in the tool.

The questionnaire was initially prepared in English and translated to Amharic. Two focus group discussions were made to validate the tool and was made to be locally appropriate. For example, local names for some substance, local interpretation of alcohol drinking, utilizations of simple and local appropriate terms were adjusted. It was also pretested among 40 students from non-participating departments and corrections on question flow and skip patterns were made after pretest. Instructors from the university were recruited and trained as data collectors while principal investigators supervise the data collection process.

Data was first cleaned manually and Questionnaire that did not fulfill minimum criteria for completeness were rejected from entry. Data entered in to Epi-Info template was cleaned and exported to SPSS version 20 for analysis. Both univariate and bivariate analysis were done. Associations were computed using odds ratio as a measure of association, with *p* < 0.05 and 95% confidence interval as level of significance.

## Result

### Socio demographic and academic characteristics of study participants

From the total sample of 695 participated, 617 students fill the questionnaire with minimum criteria for completeness giving a response rate of 89%. The mean age of participants was 21.6 years. Majority (58.7%) of participants were male, 541 (87.5%) were Orthodox Christian, and 562 (90.9%) were not married. The median monthly income was 450 birr (19.9 USD per month). Most of study participants (88.7%) completed their preparatory school at public schools. Detail data on socio demographic variables were given in Table 1.

**Table 1:** Socio-demographic characteristics of study participants, DBU June 2016

Variables	Frequency (%)
Sex	
Male	363 (58.8)
Female	254 (41.2)
Age (years, *n* = 569)	
15–19	75 (13.2)
20–24	451 (79.3)
≥ 25	43 (7.3)
Religion	
Orthodox	540 (87.5)
Muslim	29 (4.7)
Protestant	44 (7.1)
Other	4 (0.7)
Region	
Tigray	37 (6.0)
Amhara	274 (44.4)
Oromia	59 (9.6)
SNNP	31 (5.0)
Addis Ababa	136 (22.0)
Other^a^	8 (1.34)
Income Category (in birr)	
≤ 500	306 (68.8)
501–1000	92 (20.7)
≥ 1001	47 (10.6)
Mother’s Educational Status	
Not Educated	277 (44.9)
Primary school	134 (21.7)
Diploma	55 (8.9)
Degree and above	67 (10.9)
Father’s Educational Status	
Not Educated	213 (34.5)
Secondary school	71 (11.5)
Diploma	56 (9.1)
Degree and above	126 (20.4)
Preparatory School	
Public	547(88.7)
Private	63 (10.2)
NGO	7 (1.1)

^a^Other = *Harar, Dire Dawa, Somalia, Gambella, &B.Gumz*

Having the proportional allocation to the size of students per each college and year of study, 344 (55.8%) participants were from Engineering college, 175 (28.4) were first year while 388 (62.9%) were from 2nd year to 4th year students. Almost all (94.7%) study participants were using campus dormitory as a regular residency while two thirds (60.1%) of participants were using university cafeteria for regular food service.

### Student’s utilization of substances

The lifetime utilization of Alcohol, Khat and Cigarette among students was found to be 36.3%, (CI = 32.3–40.2), 10.9% (CI = 8.5–13.8) and 7.4% (CI = 5.4–9.9) respectively. The lifetime utilization of Shisha and Cannabis was 4.2% and 4.5% while ever use of cocaine or heroin was reported by only three students. In the last 30 days preceding the survey, about 16.9%, 5.7% and 3.1% of university students were utilizing alcohol, khat, and cigarette respectively. Sixty two (11.2%) students have ever used at least two of the three commonly available licit substances (alcohol, cigarette and khat). Majority of students started substance use when they were preparatory school students. Students’ substance use behavior and time of initiation of substance was given in Tables 2 and 3 respectively.

**Table 2:** Prevalence of substance use among Debre Berhan University students, June 2016

Variables	Frequency (%)
Ever drink alcohol	
Yes	224 (36.3)
No	393 (63.7)
Total	617(100.0)
Current use of Alcohol	
Yes	104 (16.9)
No	93 (15.1)
Total	197 (31.9)
Ever Chew Khat	
Yes	63 (10.9)
No	515 (83.5)
Total	578 (93.7)
Current use of Khat	
Yes	35 (5.7)
No	29 (4.7)
Total	64 (10.4)
Ever Smoke Cigarette	
Yes	42 (7.4)
No	528 (85.6)
Total	570 (92.3)
Current use of Cigarette	
Yes	19 (3.1)
No	21 (3.4)
Total	40 (6.0%)
Ever use Shisha	
Yes	25 (4.1)
No	590 (95.9)
Total	615 (100)
Ever Use Cannabis	
Yes	26 (4.5)
No	547 (88.7)
Total	573 (92.9)
Ever use Cocaine	
Yes	3 (2.5)
No	104 (97.5)
Total	107 (17.3)

*Current use = utilization of substances in the last 30 days preceding the survey*

**Table 3:** Time of Starting Substance use among DBU students, June 2016

Variables	Frequency (%)		
Time to start substance use	Drinking Alcohol	Chewing Khat	Smoking Cigarette
Before preparatory school	59 (29.2)	21 (35)	14 (34.2)
During preparatory school	69 (34.2)	22 (36.7)	15 (36.6)
1st year university	28 (13.9)	7 (11.7)	6 (14.6)
2nd year university	23 (11.4)	5 (8.3)	4 (9.8)
3rd year and above university	23 (11.4)	5 (8.3)	2 (4.9)
Mean Age to start Substance	18.5	19.2	18.5

### Perception towards substance use

Students were asked about their views towards substances use and possible reasons for utilization of substances. Majority (83.5%) did not recommend any substance for university students. Only 43 (7.8%) students agree with the statement ‘Khat improves students’ academic status’. Enjoyment (29%), relieving stress (23.7%) and peer pressure (18.3%) were commonly mentioned reasons by substance user students (Table 4).

**Table 4:** Students’ perception towards substance use and their reasons, DBU June 2016

Variables	Frequency (%)
Which substance is recommended for university students	
Cigarettes	7 (1.3)
Chat	27 (4.9)
Alcohol	6 (1.0)
None	515 (83.5)
Total	556 (90.1)
Khat improves students’ academic status	
Agree	43 (7.8)
Disagree	509 (92.2)
Total	552 (89.5)
Perceived reasons for substance use	
Individual	281 (57.1)
Family	45 (9.2)
University Administration	47 (9.6)
Teaching Learning	35 (7.1)
Environmental (Near campus)	84 (17.1)
Total	492 (79.4)
Self-Reasons for starting substance use	
Peer Pressure	17 (18.3)
To be Modern	5 (5.4)
Avoid Stress	22(23.7)
Enjoyment	27 (29)
Testing	12 (12.9)
Energy for reading	7(7.5)
Other	3 (3.2)
How substance affect your life	
Benefited	14 (15.7)
Harmed	27 (30.3)
Neutral	48 (53.93)

### Factors associated with substance use

To identify factors associated with students’ lifetime substance use behaviour, univariate binary logistic regression was done for different demographic and social variables. The computed prevalence of lifetime substance use (using at least two substances) was used as an outcome variable. Then, all variables with significant association in the univariate logistic regression were grouped in to three models (socio demographic background, university life style and social factors) and entered for further multivariate analysis.

Using the univariate binary logistic regression analysis, male than female students (COR = 3.3, CI = 1.7–6.4), age > = 30 years than adolescent (COR = 11.6, CI = 2.6–50) and those who were in private preparatory school than in public preparatory school (COR = 4.8, CI = 2.5–9) were found to be more substance users in their lifetime. Comparing with students of well-educated families (degree and above), students of uneducated fathers (COR = 0.33, CI = 0.2–0.7) and mothers (COR = 0.32, CI = 0.2–0.7) were less likely to use substances. Students from medicine (COR = 5.4, CI = 1.3–21.5) and engineering (COR = 2.3, CI = 1.1–5.73) sciences are more likely to use substances when compared to social sciences and humanities students. Furthermore, students who did not consume food in the university cafeteria were 2.2 times more likely to use substances than those who feed in the university cafeteria. Students of fourth year and above are 2.4 times more likely to use substances than first year students. Higher monthly income (COR = 6.4, CI = 3–13.4), having substance user friend (COR = 22.4, CI = 10.8–46.5), and having substance user family (COR = 8.0, CI = 3.8–16.8) were other variables significantly associated with students’ lifetime substance use behaviors. There is no significant association between substance use and religion and ethnicity.

Using the multivariate binary logistic regression, being male (AOR = 4.8, CI = 2.3–9.8), being from private preparatory school (AOR = 5.2, CI = 2.4–11.3) having higher monthly income (AOR = 5.1, CI = 1.9–13.9), and being none café (AOR = 1.8, CI = 1.1–3.2) were significantly associated with substance use. Furthermore, students who have substance user families are three times more likely to be lifetime substance users (AOR = 3, CI = 1.2–7.6) and students who have substance user friends are 24 times more substance users than their counter parts (AOR = 24.3, CI = 10.8–54.6). Multivariate regression analysis of students’ substance use behavior was given in Table 5.

**Table 5:** Cross tabulation and regression analysis of variables of student’s substance use behavior, DBU; June 2016

Variables	Frequency (%)		AOR (95% CI)
	No	Yes	
Sex			
Male	275 (84.6)	50 (15.4)	4.8 (2.3–9.8)
Female	218 (94.8)	12 (5.2)	1.00
Preparatory School			
Public	448 (91.4)	42 (8.6)	1.00
Private	40 (69)	18 (31)	5.2 (2.4–11.3)
NGO	5 (71.4)	2 (28.6)	5.8 (0.8–44)
Average Monthly Income (birr)			
< 500	255 (91.1)	25 (8.9)	1.00
501–1000	71 (85.5)	12 (14.5)	1.7 (0.8–4.1)
> 1000	27 (61.4)	17 (38.6)	5.1 (1.9–13.9)
Food Service			
Used university Café	312 (92.0)	27(8.0)	1.00
None Café (by self)	181(83.8)	35(16.2)	1.8 (1.1–3.2)
Family Use			
No	450 (91.5)	42 (8.5)	1.00
Yes	20 (57.1)	15 (42.9)	3 (1.2–7.6)
Friend Use			
No	387 (97.5)	10 (2.5)	1.00
Yes	76 (63.3)	44 (36.7)	24. (10.8–54.6)

## Discussion

This study aimed at assessing the prevalence of substance use and factors associated with substance use behavior among Debre Berhan university students. Unlike previous studies done in other Ethiopian universities, it adds information on the prevalence of illicit drug use and time of initiation of substance use. Compared to studies conducted in most of other Ethiopian universities, both lifetime and current substance use among DBU was low. Only two studies; one from Bahirdar University [16] and another study from Addis Ababa University [17] reported similar prevalence (around 7%) of khat chewing with DBU students. Prevalence of khat chewing among Axum, Hawassa, Jimma, and Haramaya, university students is all higher than the figure in Debre Berhan University [13, 15, 18, 19]. Except for Axum University, possible explanation for the low prevalence of khat chewing in DBU extends to social and environmental differences. First of all, as the university is new, substance use behaviors may not be customized as broad as the senior Ethiopian universities. Second, khat was not cultivated around Debre Berhan town that could make difficulty to access by students. Third, khat chewing was not customary by the community of Debre Berhan Town that can affect students’ perception and access to khat chewing. All this factors can contribute to the low customization of khat by Debre Berhan university students compared to other Ethiopian Universities.

As substance use are not customary in Debre Berhan Town, responses could be exposed to social desirability bias. This may cause denial of substance use by students and result in under reporting. Additionally, when surveys are done inside the teaching class room, those students with addiction behaviors may remain outside the class room because of their academic and dormitory life style. This can potentially introduce selection bias and result in lower estimate of substance use prevalence [22].

For designing target full preventive strategies, it is worth to identify the time of initiation of substance use by students. In this study, majority of students started substance use before they join the university. Intention and initiation of substance use at lower schools in Ethiopia was also described by other studies [6]. It is found important that behavioral interventions focused on improving students’ awareness, attitude and intentions should be started at lower schools like grade 11 and 12.

Being male, having higher monthly income, having substance user families, having substance user friend, eating food out of university café and being from private preparatory schools are factors found associated with higher prevalence of students’ substance use. This study is consistent with majority of studies done in Ethiopia [12–18]. Being male is believed to increase risk of substance use because of higher risk taking behavior and exposure to peer pressure among male students. Students who get food service outside the university cafeteria are more likely to use substance because of increased conditional and environmental access to substance selling houses. The association between being from private preparatory school and higher substance use rate could be attributed to the life style of private school students and their pocket income but it needs further explanation by others studies.

This study indicated that students with better monthly income (pocket money) are more likely to use substances and is reported in other studies in Ethiopia and [18] other countries [22–25].

A crosssectional survey conducted among school children in North West England indicated that binge, frequent and public drinking of school adolescents was strongly related to amounts of spending money youths have available [23]. A longitudinal, nationally-representative survey of secondary school students in the United States showed that higher parental education and household income were significantly associated with higher odds of binge drinking, marijuana use and cocaine use [24]. Another study among United States Universities also reported that spending money amount was significantly positively associated with likelihood of getting drunk [25]*.*

This association could be explained by the fact that students who have better monthly pocket money will have easily access to purchase substances. As was noted earlier, majority of university students in Ethiopia are living far from parent’s direct control while receive money from their parents. Therefore, this find suggested that parents should minimize or regulate the amount of pocket money sent to their students who are living far to them. It may be also vital to increase the cost of substances to reduce access to alcohol and thus consumption.

Having substance user friends and families are most likely to increase substance use behaviors because they let students to familiarize substances and adopt utilization thereby reducing the subjective norm and perceived risk perception of students. This finding indicates that interventions to reduce substance use among youth should consider not only individual factors but also other social and environmental factors.

This study is able to report the prevalence of both licit and illicit drugs used by university students. Its comprehensive assessment of multiple factors associated with substance use behavior is conserved as strength. The inclusion of unavailable substance ‘Relevin’ in the survey and its response by only one student indicates that there is low potential over reporting of drug use. Before interpreting findings, readers should consider limitations to the methodology and field work of this research. First, there is no validity assurance for underreporting of substance use and students are more likely to deny their utilization behavior because of social desirability bias. Second, even though we used stratified two stage sampling with proportional allocation, we didn’t analyze using the complex analysis method and standard errors were not estimated for weighted samples.

## Conclusion

The prevalence of substances use among DBU students is low comparing to other Ethiopian and African universities. There is a need for further research with more powerful sample size and weighted estimates using complex analysis. Reasons for lower prevalence of substance use from other Ethiopian universities shall be further explored using qualitative study. Youth are starting substance use at lower grades especially at preparatory schools. Lifetime substance use behaviors are affected by complex factors at individual, family, school, social, and environmental factors. Therefore, strategies to alleviate youth substance use problems should focus on changing individual perception, knowledge, and intention towards substances. Students should be trained on life skills so that they can cope with peer pressures. Families should be role model to their children by avoiding substance use and is recommended to manage the pocket money they sent to their children. Other interventions focusing on reducing access to substance should be implemented at federal, local and university level.
